# Surgical treatment of adolescent idiopathic scoliosis: Complications

**DOI:** 10.1016/j.amsu.2020.02.004

**Published:** 2020-02-24

**Authors:** Omar A. Al-Mohrej, Sahar S. Aldakhil, Mohammed A. Al-Rabiah, Anwar M. Al-Rabiah

**Affiliations:** a Department of Orthopedic Surgery, King Faisal Specialist Hospital & Research Centre, Riyadh, Saudi Arabia; b Department of Orthopedic Surgery, King Abdulaziz Medical City, Riyadh, Saudi Arabia; c College of Medicine, Alfaisal University, Riyadh, Saudi Arabia

**Keywords:** Complications, Fusion, Idiopathic, Scoliosis, Spine, AIS, adolescent idiopathic scoliosis, DIC, disseminated intravascular coagulation, NIS, National Inpatient Sample, PJK, proximal junctional kyphosis, POVL, perio-perative visual loss, SSI, surgical site infection, VTE, venous thromboembolism

## Abstract

Despite the fact that spinal surgeries for adolescent idiopathic scoliosis (AIS) result in good outcomes for most patients, they are not without complications either medically or surgically. Neurologic injury represents the most severe complication and is, as such, the most feared. Further complications include dural tears, peripheral neuropathy, surgical-site infections, implant-related issues, thromboembolic events, visual loss, pseudarthrosis, Crankshaft phenomenon, flatback phenomenon, proximal junctional kyphosis, and mortality. It is vital that all spine surgeons to be fully conversant with the possible complications and the proper responses for each of them.

## Highlights

• Surgeons should know how to manage complications of surgery for AIS.
• Patients should know about potential complications prior to surgery.
• Neurologic injury represents the most severe complication.

## 1 Introduction

Adolescent idiopathic scoliosis (AIS) is among the most frequent deformities in children, adolescents, and young adults, with an overall prevalence of 0.47%–5.2% [1,2].

Over the past 15 years, the volume of AIS surgeries has increased significantly. In 1997, there were 1783 such surgeries; by 2012, this had increased by 193%, with 5228 surgeries recorded [3]. Posterior fusion is thought to be the most frequently used surgical approach (75%), followed by anterior and combined approaches, at 18% and 7%, respectively [4].

## 2 Complications

Despite modern instrumentation, new surgical techniques, and new technology result in good surgical outcomes, complication rates, as indicated by surgeon reports, have remained somewhat constant. Between 5% and 23% of all AIS patients experience surgical complications [3,[5], [6], [7]]. This report showed that anterior, posterior and combined procedures had complication rates of 5.2%, 5.1% and 10.2%, respectively. Another study reported complication rate of 22.3% [3]. Using the National Inpatient Sample database (NIS), Menger et al. reported postsurgical complications following AIS surgical correction of 2.8% for cardio-pulmonary complications, 3.5% for abdominal complications, 0.9% for neurological complications, 0.4% for infectious complications, 0.1% for thromboembolic events, 0.1% for renal complications and a mortality rate of 0.1% [8]. The incidence of these complication might be related to several factors, including patient and surgeon factors, making risk stratification somewhat burdensome. Nevertheless, recognizing the complications and their risk factors is imperative both for surgeons and their patients.

### 2.1 Neurological injury

Within the scope of scoliosis surgery, neurological injury is the complication that generates the most fear. Such injuries can cause a range of complications, from transient neuropraxias related to body position to spinal cord damage that causes total paralysis.

Neurological injury rates have ranged between 0.3% and 4% [9]. It has been shown that neurological injury occurred in 0.69%–1.06% of AIS spine fusion cases and varied according to the surgical approach employed [10,11].

It is crucial that surgeons understand the causes of neurologic injury and the possible types of complication. They should also know how to systematically manage any notable neurological changes during the intra-operative phase. Mid-procedure intra-operative nerve root or spinal cord injuries can occur for a number of reasons.

A surgical implant or instrument could damage the spinal cord, intra-operative hypotension could cause lower perfusion and cord ischemia, or deformity corrections could overstretch the spinal cord. Other causes of neurologic complications include epidural hematomas or abscess, and injury to nerve roots. Timely diagnosis of such injuries has been improved using neurophysiological detection of impending spinal cord injuries [12].

Vitale et al. found that electrophysiology had a specificity of 88% and sensitivity of 100% for detection of neurological deficits [13]. In each case of a true neurological event, the patient always suffered identifiable precipitating events to which the changes could be attributed. In most of these cases, the complications were effectively reversed. Intra-operative neuro monitoring should be performed in a controlled, systematic fashion so as to mitigate any risk of neurological damage.

While most neurological injuries in AIS surgery occur intra-operatively, the onset of spinal cord injuries can be delayed. According to Auerbach et al., delayed neurologic deficits have a frequency of around 0.01% [14]. This illustrates the importance of post-operative serial neurological examinations for AIS patients irrespective of their previous post-operative neurologic results.

For delayed neurological deficit, pre-operative spinal imaging (CT scans or MRIs) could be useful, allowing surgeons to accurately pinpoint the source of any compression or the location of any anomalous implant requiring revision. Accordingly, almost 75% of affected patients underwent radiological studies before repeat surgeries [14].

Predicting long-term outcomes after scoliosis surgery-caused neurologic deficits is challenging. Coe et al. reported complete recovery in 11 cases (61%), while six patients made an incomplete recovery (33%) [15]. The timing of these injuries (intra-operative versus delayed) are nevertheless unclear.

In cases where patients suffered compression-related delayed-onset neurologic deficits, there was a considerably better chance of neurological recovery when compared to whose deficits were caused by ischemia [14].

### 2.2 Dural tears

In AIS surgeries, dura exposure and subsequent dural tears are uncommon because these are relevant only in cases where the deformity is either rigid or severe. Dural tears in AIS surgeries have a frequency of between 0.12% and 0.26% [6]. Such tears should be repaired in timely manner. In our experience, if a patient suffers a dural tear and a repair is made, we limit the mobilization of the patients during the post-operative and sometimes we place a lumbar drain.

### 2.3 Position-related complications

It is important that surgeons fully understand the proper positioning of spinal fusion patients. Typically, in posterior AIS fusions, patients are positioned prone. In terms of patient positioning, complications include brachial plexus injuries resulting from abduction of upper extremities [16]. Peripheral nerves palsy results from direct compression on an operating room table. The frequency for such complications is around 0.5% [6].

As such, surgeons, anesthesiologists and circulating nurses must make every effort to ensure that patients are appropriately positioned and that their bony prominences are padded. Doing so mitigates frequent, preventable complications such as ophthalmologic injuries, peripheral nerve neuropraxia and brachial plexopathy.

### 2.4 Visual loss and ophthalmic injury

Perioperative visual loss (POVL) is one of most devastating complications one might face after scoliosis surgeries, although it is incredibly rare. Nevertheless, Myers et al. reported 37 instances of ophthalmological complications [17]. In another study by Ramos et al., POVL after scoliosis surgery had a rate of 0.16% [18]. Increased risk was associated with young patients, anemic patients, and long-segment fusions.

### 2.5 Intra-operative bleeding

Spine surgeons are familiar with the fact that surgical interventions for AIS patients is generally correlated with intra-operative bleeding that necessitates transfusion [19]. Risk factors include female gender, greater Cobb angle, low pre-operative hemoglobin levels, fusions of longer segments, and lumbar spine fusions [19,20]. A Study found that around 25% of all AIS patients received transfusions [21]. There is no clear literature on the negative consequences of blood transfusion for AIS patients. However, several strategies are being used perioperatively to decrease the need of blood transfusions [22].

#### 2.5.1 Gastrointestinal (GI) complications

During the post-operative period after AIS surgery, GI issues are common, as they are after any other major orthopedic surgery. Menger et al. found that, of 75,106 spinal procedures for AIS, 2.7% caused post-operative GI morbidity [8]. Risk factors for GI morbidity include extensive opioid use, medical comorbidities and fusion procedures. To reduce these risks, multiple interventions are used, including early post-operative mobilization, post-operative epidurals, and multimodal non-narcotic pain control medications.

### 2.6 Infection and wound complications

In AIS patients, Surgical site infection (SSI) rates range from 0.17% to 9%, according to patient demographics [23]. SSI rates were between 0.17% and 1.37% in an SRS database series [6,15]. Considering all idiopathic cases, the authors found 0.5% and 0.8% of superficial wound infection and deep wound infections, respectively.

Risk factors include non-idiopathic scoliosis and pelvic instrumentation, as per Mackenzie et al. [24], *Staphylococcus aureus*, *coagulase-negative staphylococci*, and *Pseudomonas aeruginosa* were the most common pathogens. In the majority of cases, AIS patients were found to experience delayed-onset infections, with such infections occurring more than 6 months after the procedure [25,26]. The authors also suggested that acute post-operative deep infections were more infrequent in AIS than in non-idiopathic scoliosis. While neuromuscular patients were more commonly affected by highly virulent gram-negative bacteria, most delayed SSIs in AIS patients resulted from low virulence skin flora [27].

Potential risk factors for such delayed SSIs are likely to be caused by a number of factors such as significant medical histories and blood transfusions [26].

The management of early-onset infections should include every effort to irrigate and debride the wound, leaving the implants in-situ wherever possible. This treatment should be combined with infectious disease specialist consultations and a long-term course of antibiotics. The available data do not indicate a specific recommended length for the antibiotic treatment. As such, the ideal duration for this antibiotic treatment is highly subjective.

When patients suffer from wound dehiscence and deep infection, it may be useful for their treatment to include serially-applied closed negative pressure dressing systems. Generally, such infections are appropriately managed with antibiotics, irrigation and debridement (I&D), and hardware removal.

A study conducted by Li et al. discussed the efficiency of oral antibiotic therapy for orthopedic infections including the spine [28]. The authors reported that oral antibiotics were similarly effective to intravenous (IV) antibiotic therapy in the period of first 6 weeks. Surprisingly, oral antibiotic therapy was associated with shorter hospital stays and with fewer complications than was IV therapy [28].

With respect to implant removal, Di Silvestre et al. reported that patients with delayed-onset infections of after fusion surgery required implant removal to completely treat the infection [29]. It has been shown that, when an implant is removed, deformity progression is only modest [29]. Furthermore, any continued progression can be successfully treated when the patient is free from infection.

Despite the fact that infection is rare among AIS patients, surgeons should refer to best practice guidelines when creating their own strategies for SSI prevention.

### 2.7 Thromboembolic events and pulmonary complications

Venous thromboembolism (VTE) is a widely recognized complication of spine surgeries, in adults. VTE is relatively infrequent within pediatric scoliosis surgeries with a rate of 21/10,000 [30]. Risk factors include syndromic and congenital scoliosis along with spine fusion surgery after a fracture and greater patient age. It is likely that this clinical series underrated the true frequency of VTE, because it only included events that happened in-hospital. VTE can occur weeks to months after surgery. There are presently no official recommendations to guide surgeons regarding perioperative prevention for VTE in AIS patients or, indeed, its treatment.

Pulmonary complications, unrelated to embolus, are poorly characterized, although they are among the most frequent complications after AIS spine surgery. Such complications have a reported incidence of between 0.6 and 3.5% [3]. There is an association with prolonged posterior surgeries and anesthesia. There are conflicting reports as to whether or not these post-operative pulmonary issues have a direct correlation with pre-operative pulmonary function tests or spinal curve magnitudes [31,32].

### 2.8 Implant-related issues

In AIS spinal surgeries, implant-related issues have been shown to represent between 0.64% and 1.37% of all complications [15]. Such issues include those caused by rods, hooks, or screws. While studies have shown pedicle screws usage to be safe and effective for AIS patients [33], numerous reports refer to issues caused by pedicle screw placement [34]. Complications include screw loosening, wear debris, neurological issues, dural tears, wound complications, pleural effusion and pneumothorax [35].

### 2.9 Other co-morbidities

Major complications, including shock, disseminated intravascular coagulation (DIC), myocardial infarction, cardiac arrest, neurological injury, malignant hyperthermia, and iatrogenic stroke, had an incidence of 0.2% [36]. Nevertheless, the main drawback of this study was that the NIS database comprises in-patient data; therefore, complications arising after discharge were not studied.

### 2.10 Pseudarthrosis

The pseudarthrosis rate for AIS patients has declined to around 1% because of improved surgical techniques and instruments. A systematic literature review found that pseudarthrosis occurred in 22.7§%, with a rate of 2%–7% for instrumented fusions [36].

Pseudarthroses were found to occur in 2% of cases in a meta-analysis of 1565 instrumented posterior spinal fusions; instances were less common with Cotrel-Dubosset constructs (2%) than with Harrington rods (3%). Pseudarthrosis was not seen in the patients with pedicle screw fixation [5]. Pseudarthrosis is usually detected using bone scans, computed tomography, or oblique radiographs; however, definitive diagnosis can only be obtainable during the surgical exploration. When there is a need for surgery, in cases with pain, pseudarthrosis is approached in the same way as any other joint needing fusing: edges undergo freshening and decortication and a bone graft is used, followed by use of the usual instrumentation.

### 2.11 Curve progression

One of most frequent causes of revision, with a rate of around 1%, is curve progression. Luhmann et al. [37], having undertaken 1057 AIS fusions, found reoperation was necessary for 4% of patients, the majority of which were for pseudarthrosis (26%) or infection (34%). Around 2% of the patient sample (17% of reoperations) were necessary because of curve progression.

Progression of the curve below the spinal fusion level is known as the “adding-on” phenomenon [[38], [39], [40]]. Reports have quoted rates between 2% and 5% of subjects. Cho et al. [38] demonstrated that notable predictors of adding-on were age and Risser grade. Furthermore, the chances of adding-on were doubled when L4 was tilted to the right side.

The clinical effect of adding-on remains unclear; however, the frequent requirement for reoperation and other and satisfactory clinical outcomes have been noted [41,42].

### 2.12 Crankshaft phenomenon

The crankshaft anomaly occurred relatively frequently in previous posterior instrumentation systems; nevertheless, the frequency has declined notably recently, mainly due to the employment of “growth-friendly” techniques [5]. The crankshaft phenomenon may arise if notable growth is remaining because of continual anterior growth despite posterior spinal fusion. This happens more frequently with children of 10 years of age or less with a Risser stage of 0 or 1, and an open triradiate cartilage; it occurs relatively infrequently in adolescence, when the subjects are closer to skeletal maturity [5].

With younger patients, it may be desirable for surgery to be postponed, and anterior fusion should be taken under consideration with those most likely to suffer the crankshaft phenomenon.

### 2.13 Flatback deformity

Flatback deformity and decompensation occur much less frequently with the spine instrumentation presently available than they did when Harrington rods were commonly employed [43]. Decompensation risk factors include crankshaft phenomenon, improper fusion levels, rigid lumbosacral hemicurve, overcorrection for the thoracic curve, failure to identify curve patterns, derotation, curve progression at the lumbar spine post thoracic fusion, and the adding-on to the fused spine distally or proximally. Depending on the remaining quantity of growth, management of the compensation can be undertaken by extending the fusion or orthosis [5].

### 2.14 Proximal junctional kyphosis

Proximal junctional kyphosis (PJK) is a deformity of the sagittal plane observed following instrumented fusion for AIS [39]. For AIS patients, this frequently manifests as a kyphotic deformity that is reported in 17%–39% of the cases. Risk factors that have been reported include a surgical correction for thoracic kyphosis over 50, types of instrumentation, and fusion to the sacrum. Helgeson et al. evaluated 283 AIS patients and found PJK prevalence of 0%, 2.5%, 5.6%, and 8% with hooks-only instrumentation, hybrid instrumentation, screws/proximal hooks, and all-screws constructs, respectively [44]. Nevertheless, PJK appears to have minimal influence on surgical outcomes [44,45].

## 3 Conclusion

For patients with AIS, spinal fusion surgery offers a means of deformity correction that is both effective and relatively safe. Surgeons report complications in between 5% and 25% of cases. It is important for surgeons, specialists, and residents who treat AIS patients to be fully conversant with the nature and management of potential complications. Furthermore, it is important to inform the patients of the potential complications before surgical intervention is commenced.

## Ethical approval

Not applicable.

## Sources of funding

There are no source of funding to declare.

## Author contribution

Study Concept: Anwar M. Al-Rabiah and Omar A. Al-Mohrej

Writing the paper: Omar A. Al-Mohrej, Sahar S. Aldakhil, Mohammed A. Al-Rabiah, Anwar M. Al-Rabiah.

All of the authors are accountable for all aspects of the work and they have revised the final version of this manuscript.

## Research registration number

Name of the registry: Not applicable.

Unique Identifying number or registration ID: Not applicable.

Hyperlink to the registration (must be publicly accessible): Not applicable.

## Guarantor

Omar A. Al-Mohrej

## Consent for publication

Not applicable.

## Provenance and peer review

Not commissioned externally peer reviewed.

## Declaration of competing interest

None of the authors have any conflict of interest to report.
